# The effect of exercise training level on arterial stiffness after clinically significant weight loss

**DOI:** 10.1111/cob.12584

**Published:** 2023-02-27

**Authors:** Damon L. Swift, Joshua E. McGee, Emily E. Grammer, Anna C. Huff, Marie C. Clunan, Nicole Hursey, Taylor T. Brown, Briceida G. Osborne, Joseph A. Houmard, Robert A. Carels, Walter J. Pories, Laura E. Matarese

**Affiliations:** 1 Department of Kinesiology, East Carolina University, Greenville, North Carolina, USA; 2 Human Performance Laboratory, East Carolina University, Greenville, North Carolina, USA; 3 ECU Health Wellness Center, Greenville, North Carolina, USA; 4 Department of Psychology, East Carolina University, Greenville, North Carolina, USA; 5 Department of Surgery, East Carolina University, Greenville, North Carolina, USA; 6 Department of Internal Medicine, East Carolina University, Greenville, North Carolina, USA

**Keywords:** arterial stiffness, exercise, obesity, pulse wave velocity, weight loss, weight maintenance

## Abstract

Arterial stiffness is improved by weight loss. However, no data exist on the impact of aerobic exercise levels on arterial stiffness during weight maintenance. Adults who were overweight or with obesity (*N* = 39) participated in a 10-week weight loss program. Participants who achieved ≥7% weight loss were randomized to aerobic training at the minimum physical activity guidelines (PA-REC, 550 MET min/week) or weight maintenance guidelines (WM-REC, 970 MET min/week) for 18 additional weeks. Arterial stiffness (carotid-to-femoral pulse wave velocity [cfPWV], augmentation index normalized for 75 beats/min [AIX75]) and blood pressure [aortic and brachial]) were assessed at baseline, the end of the weight loss phase (week 10), and follow-up (week 28). There was a reduction in cfPWV in participants who met the weight loss goal (−0.34 m/s, *p* = .02) and approached significance for the entire sample (*p* = .051). Similarly, there were reductions in AIX75, brachial blood pressure, and aortic blood pressure (*p* < .05) in the full sample. In the weight maintenance phase, no differences were observed between the PA-REC and the WM-REC groups for change in arterial stiffness or blood pressure (*p* > .05). However, changes in cfPWV were independently associated with changes in LDL (*r*^2^: 0.45, *p* = .004) and exercise intensity (*r*^2^: 0.17, *p* = .033). Aerobic exercise level at the minimum physical activity guidelines or weight maintenance guidelines does not affect the change in PWV or the change in cfPWV after clinically significant weight loss. However, interventions which limit increases in LDL cholesterol and promote high-intensity aerobic exercise may prevent increases in stiffness during weight maintenance.

## 1 ∣ INTRODUCTION

Obesity is associated with an increased risk of hypertension,^1,2^ cardiovascular disease (CVD),^3-5^ and other cardiovascular risk factors.^6^ Elevated arterial stiffness, an underlying factor in the pathophysiology of hypertension, is associated with endothelial dysfunction, vascular remodelling, increased sympathetic drive and elevated cardiovascular mortality risk.^7-12^ The consequences of increased vascular stiffness on the heart include increased afterload, cardiac-metabolic demand, and risk of left ventricular hypertrophy.^13^ Obesity is comorbid with the presence of other traditional clinical risk factors (e.g., impaired glucose tolerance,^14,15^ dyslipidemia,^16,17^ low cardioresporatory fitness,^18,19^ and elevated systemic inflammation^20^), which are associated with increased arterial stiffness. Therefore, interventions that reduce arterial stiffness have important clinical implications for improving vascular health in adults who are overweight or with obesity.

Clinically significant weight loss (5%–10%) has been shown to improve carotid-to-femoral pulse wave velocity (cfPWV),^21,22^ which is the gold standard measurement of arterial stiffness.^13^ Chronic aerobic exercise training improves arterial stiffness independently of weight loss, which may be due to improvements in endothelial function, autonomic control (reduced sympathetic tone and increased parasympathetic tone), or reductions in neurohumoral vasoconstrictors.^23^ A recent meta-analysis observed that lifestyle interventions improve cfPWV by 0.8 m/s,^21^ which is associated with an ~11% improvement in CVD risk based on epidemiological data.^24^ While both weight loss and exercise can independently improve cfPWV, little data are available on the combined effects of diet and exercise on cfPWV. Additionally, the data from combined approaches of weight loss and exercise have generally not been supervised exercise or strictly controlled, where exercise metrics could be evaluated as potential mediators of response (e.g., exercise time, intensity, etc.) of cfPWV. Further confirmation of the independent factors associated with the improvement of arterial stiffness from weight loss is necessary to design effective lifestyle-based interventions.

Similarly, little data are available on the impact of aerobic exercise on factors associated with cfPWV during weight maintenance.^25^ This is important given that regression in weight after clinically significant weight loss is associated with increased CVD risk factors and worsened hypertension outcomes,^26,27^ while cardiorespiratory fitness^19^ and exercise training duration^28^ are associated with improved pulse wave velocity. Moreover, a dose–response relationship has been observed between exercise amount and improvement in cardiorespiratory fitness.^29^ Therefore, it is possible that the aerobic exercise training level that is recommended for weight maintenance (>200 min of moderate aerobic physical activity) can preserve improvements in arterial stiffness after clinically significant weight loss to a greater extent than a lower amount of exercise. To our knowledge, no studies have directly addressed this issue.

The purpose of the present study was to evaluate the effect of weight loss (via caloric restriction) combined with supervised aerobic exercise training on arterial stiffness, blood pressure, and other vascular outcomes in participants who are overweight and with obesity from the PREVAIL-P study. We also examined the impact of the aerobic exercise equivalent to the weight maintenance guidelines versus the minimum public health guidelines for 18 weeks following clinically significant weight loss on cfPWV and other relevant arterial measures.

## 2 ∣ MATERIALS AND METHODS

The present investigation is an ancillary study from the Prescribed Exercise to Reduce Recidivism after Weight Loss pilot (PREVAIL-P) study. The study was approved by the East Carolina University (ECU) institutional review board and registered on ClinicalTrials.gov (NCT03685123). A detailed description of the methodology of PREVAIL-P has been previously published.^25^ In brief, we enroled 39 individuals who were sedentary, overweight (BMI: 25–29.9 kg/m^2^) with one additional cardiometabolic risk factor (e.g., dyslipidemia, hypertension, etc.) or with obesity (BMI: 30–39.9 kg/m^2^). Participants were excluded if they had type 2 diabetes, significant cardiovascular disease, excessively high systolic (>160 mmHg) or diastolic blood pressure (>90 mmHg), had previous weight loss surgeries or a major health condition that were contraindications for weight loss or exercise training. In addition, we excluded individuals who were taking medications or had conditions that could confound weight loss or regain (e.g., hypo/hyperthyroidism, weight loss medication, etc.).

Participants were recruited through emails to ECU employees, screening through a study website, flyers in local physician offices, and from a local weight loss clinic. After initial eligibility was determined, a screening visit for the study was scheduled. At the first screening visit, the research coordinator provided information to each participant about the study, research procedures; potential risks and benefits, and answered any remaining participant questions. If the individual was interested in participating, the consent form for the study was signed. The study was approved by the ECU University and Medical Center Institutional Review Boards. Research staff then assessed the participant for the full study inclusion criteria, obtained demographic and contact information and measured the individual's height, weight, and resting blood pressure to confirm eligibility. The participant was also screened for major barriers to study completion (e.g., distance from home to the facility, available time for participation, weekly time commitments, and acceptability for participation in both weight loss and exercise aspects of the study). A research nurse drew a blood sample following a 12-h fast to evaluate hepatic, renal, haematological, endocrine, and metabolic function for inclusion/exclusion purposes. Blood lipids, glucose and insulin levels were also measured. Premenopausal women were required to take a pregnancy test to confirm that they were not pregnant. Laboratory results were subsequently reviewed and approved by the study physician. Following the completion of screening measures, the participant was scheduled for baseline testing.

Primary and secondary outcome measures were assessed at two separate visits. Both visits were conducted in a fasted state (12 h). During the first visit, we obtained body weight, body composition, and cardiorespiratory fitness testing data. On a separate day, we measured pulse wave velocity, augmentation index, and obtained blood samples.

### 2.1 ∣ Outcome measures, visit 1

#### 2.1.1 ∣ Body composition

Body composition was measured using whole body dual-energy x-ray absorptiometry (DXA) (Hologic, Horizon A Marlborough, MA) with the participant in the supine position. From the whole body DXA scan, we quantified total fat mass, visceral fat mass (at the level of L4), lean tissue mass, and bone mass (APEX version 5.6.0.5). Waist circumference was evaluated with a Gulick tape measure at the natural waist. Three measurements were recorded in centimeters and the results were averaged.

#### 2.1.2 ∣ Maximal exercise test

A modified Balke treadmill (Trackmaster 425, Carefusion, Newton Kansas) protocol was used to determine cardiorespiratory fitness and the appropriate heart rate range for aerobic exercise training. During the test, participants walked at an initial speed of 2.0 mph with a 0% grade for the first 2 min, after which the treadmill speed was increased to 3.0 mph for the next 2 min. Treadmill grade was increased by 2.5% every 2 min until volitional exhaustion. Respiratory gases (VO_2_, CO_2_) and ventilation were measured continuously using a True Max 2400 Metabolic Measurement Cart (Parvomedics, Salt Lake City, Utah). Fitness was quantified in relative (ml/kg/min) and absolute terms (L/min) and estimated metabolic equivalents (METs).^30^

### 2.2 ∣ Outcomes measures, visit 2

#### 2.2.1 ∣ Blood measures

A fasting blood sample was obtained via venipuncture for analyses of fasting lipids (e.g., total cholesterol, high-density lipoprotein, low-density lipoprotein, very low-density lipoprotein), glucose, and insulin values. The sample was subsequently sent to LabCorp (Burlington, NC) for analysis using standard analytic techniques.

#### 2.2.2 ∣ Pulse wave velocity and wave reflection properties

cfPWV and aortic blood pressure parameters were measured using a SphygmoCor XCEL (AtCor Medical, Sydney, Australia). Testing was performed in a quiet, temperature-controlled room (70 F°) in the morning. Prior to testing, participants refrained from large meals and caffeine for at least 2 h and refrained from alcohol, vigorous exercise, and vasoactive medication for at least 12 h, in accordance with established guidelines.^13^ Aortic blood pressure parameters (e.g., brachial blood pressure, aortic blood pressure, augmentation index normalized for 75 beast/min [AIX75]) were obtained in the seated position after a 5-min rest. After this, the participant rested in the supine position on a hospital bed for 15 min and a blood pressure cuff was placed on the mid-thigh. Study staff measured the distance from the sternal notch to the cuff, from the carotid artery to the sternal notch, and from the femoral artery to the cuff using a tape measure. Following this rest period, a tonometer was held against the carotid artery during the measurement. Once adequate pulse waveforms were visible, we measured cfPWV during 10-s intervals. cfPWV was quantified in meters per second (m/s). Then, a second cfPWV measurement was conducted. If the first two measurements were ≤0.5 m/s of each other, we reported the average of both measurements. If the difference between measurements was >0.5 m/s, a third measurement was performed and the median of the three measurements was used to quantify cfPWV.^13^ The same investigator performed each measurement on the participant at all three time points.

#### 2.2.3 ∣ Intervention

After baseline measurements were obtained, all participants began the weight loss phase of the trial in a rolling fashion. The weight loss phase included an OPTIFAST weight loss program (ECU Health Wellness Center, Greenville, NC) and supervised aerobic exercise training at ECU. The goal of the weight loss program was to induce clinically significant weight loss (defined as ≥7% based on procedures from major weight loss trials) and other studies.^31-34^

#### 2.2.4 ∣ OPTIFAST^®^weight loss program

OPTIFAST is a comprehensive, medically supervised weight loss program that combines lifestyle education and medical monitoring with portion-controlled, nutritionally-balanced meal replacement products (e.g., shakes, bars, and soups). The major goal of the OPTIFAST program was to provide clinically significant weight loss equal to or exceeding 7% weight loss. During their enrolment in the OPTIFAST program, participants also performed supervised exercise training (exercise methodology described below).

Participants first received a nutrition assessment with a registered dietitian nutritionist. The active weight loss phase of the program consisted of 10 weeks, with the first 8 weeks consuming full meal replacement. Each OPTIFAST product contained 160–170 calories, 14 g protein (whey, casein, and/or soy), 3 g total fat, 0 *trans*-fat, ~20 g carbohydrate, 220 mg sodium, 470 mg potassium, <1 g lactose, and 10%–30% of the RDI for vitamins and minerals. Participant nutrient goals were based on thier baseline BMI. Participants were asked to consume approximately 5 OPTIFAST products per day (800–820 calories/day; protein 70 g/day). At week 8, participants could eliminate two products per day and introduce 350 Calories of food from a Healthy Food Exchange list. During this time, caloric intake usually increased to approximately 1300–1500 calories per day. By week 8, participants transitioned, at an individual pace, to all self-prepared food, except for 1–2 products daily, if desired. During the entire weight loss intervention, participants were instructed to log all OPTIFAST products and other foods consumed into the MyFitnessPal smartphone or computer app to help them track caloric consumption. The cost of the weight loss program and the OPTIFAST products were provided by the study's resources to help recruit a generalizable sample and to include participants from socioeconomic groups who otherwise could not afford the program. Nestle had no role in the funding or design of the project.

The overall goal of the behavior change classes was to assist participants in meeting the 7% weight loss goal and to increase compliance with the dietary aspects of the weight loss program (e.g., eating cues, motivation to change, mindful eating, etc.). Classes were delivered in a rolling fashion (i.e., participants could enter the classes at any week and go through the full 10-week progression), which allowed the assessment schedule to be feasible when participants complete the weight loss component. At each class, participants were weighed, completed a questionnaire (regarding the number of products consumed, fluid intake, and any physical changes) and received a didactic lecture on a topic relevant to weight loss.

#### 2.2.5 ∣ Exercise training during weight loss phase

Participants completed 2–3 supervised aerobic training sessions per week. The purpose of the exercise was to facilitate a greater magnitude of weight loss when combined with caloric restriction^35^ and was expected to increase the likelihood of achieving clinically significant weight loss. Additionally, the exercise prepared participants to have adequate fitness to exercise at the required levels in the weight maintenance phase of the study. The initial exercise level was 300 MET minutes per week and increased by 50 MET minutes per week until the participant reached the full amount of exercise during the initial weight loss component of 700 MET minutes (weeks 9–10).

The weekly MET minute requirements were divided into 2–3 sessions per week depending on participant preferences. Following a 5-min warm-up, participants exercised at the heart rate range associated with 50%–75% of peak VO_2_ (determined from baseline exercise testing). Heart rate was monitored and collected continuously (every second) during exercise with Zephyr Bioharness three monitors (Medtronic Annapolis, MD). Exercise adherence was quantified as weekly MET minutes exercised divided by the amount required. Exercise compliance was quantified as the number of sessions of exercise attended divided by the total number of sessions required. Aerobic exercise intensity (%VO_2_) for the weight loss phase was estimated using the heart rate/VO_2_ relationship,^36^ established from baseline exercise testing. This relationship was updated using exercise testing data from the assessments after the weight loss phase for the weight maintenance phase of the study. If participants missed an exercise session, an exercise session could be replaced in subsequent weeks (e.g., adding minutes to a subsequent exercise session or adding an additional session).

Outcome measures after the weight loss period were conducted at the end of week 10. cfPWV and augmentation index measures were conducted approximately 24 h following the last exercise session. If this could not be achieved due to scheduling issues, an exercise session was provided 24 h prior to the assessment of outcome measures (at the same amount and duration as their regular exercise sessions).

#### 2.2.6 ∣ Randomization

After the completion of the weight loss phase, only participants who obtained the 7% weight loss goal progressed to the weight maintenance phase. The randomization was conducted by the study biostatistician to either: (1) exercise levels consistent with physical activity recommendations (PA-REC) or (2) a weight maintenance recommendations group (WM-REC) for an additional 18 weeks in a 1:1 ratio. Group randomization was stratified by percent weight loss during the weight loss phase and the participant's baseline BMI.

#### 2.2.7 ∣ Exercise during the weight maintenance phase

Full details on the design and methodology for the weight maintenance phase are discussed in a methods paper.^25^ In brief, the exercise volume of the PA-REC group corresponded to the minimum amount of recommended exercise (150 min of moderate physical activity per week) based on current guidelines to improve cardiometabolic risk factors.^37^ The WM-REC group were prescribed an exercise level of 970 MET minutes per week, which translates to about 250 min per week of moderate aerobic exercise training. Thus, the prescribed amount of exercise in the WM-REC group was consistent with weight maintenance guidelines for exercise training (200–300 min per week of moderate-intensity exercise).^38^ The PA-REC group began the weight maintenance phase at 550 MET minutes per week and continued at this exercise level for the remainder of the study. The WM-REC group began the weight maintenance phase at 750 MET minutes per week and the weekly exercise level was increased by 50 MET minutes every week until the required exercise level of 970 MET minutes per week was reached (week 18). Participants in the WM-REC group continued exercising 970 MET minutes per week for the remainder of the study (weeks 19–28). The required number of exercise sessions during the weight maintenance phase was approximately 3–5 sessions per week depending on the amount of the required exercise and participant preferences.

#### 2.2.8 ∣ Sample size

**FIGURE 1:**
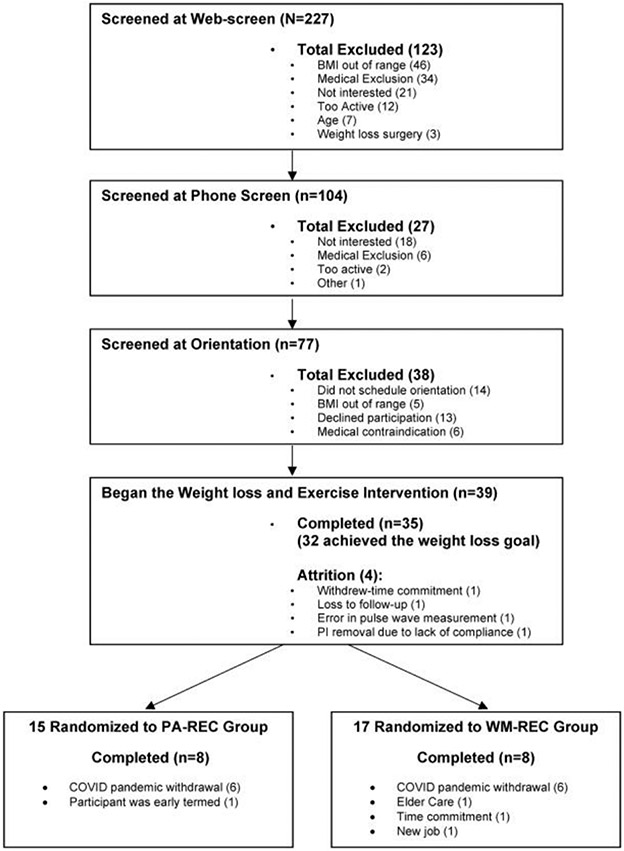
Consort diagram.

The consort diagram for the present analysis is shown in Figure 1. We included 35 participants for the weight loss phase (three others did not complete the weight loss intervention and one measurement error) of which 32 individuals achieved the weight loss goal of at least ≥7%. In the weight maintenance phase of the study, 32 people were randomized to PA-REC and WM-REC groups, respectively. At the end of the weight maintenance phase, 16 individuals completed the study (PA-REC [*n* = 8] and the WM-REC [*n* = 8]). Importantly, 12 withdrawals occurred due to the coronavirus pandemic.

#### 2.2.9 ∣ Statistics

Baseline data were summarized in means (standard deviations). Categorical variables are represented as a percentage (*n*) in each group. The primary outcome variables was the change in cfPWV. Major secondary variables included changes in AIX75, aortic blood pressure, resting blood pressure, vascular age, and cardiometabolic variables in the intervention. Pearson correlations were used to evaluate the relationship between baseline arterial stiffness factors and other cardiometabolic variables. A paired samples *t*-test was used to evaluate the change in the outcome variable from baseline to the end of the weight loss phase in the entire study sample (*n* = 35) and in a sub-sample of participants who obtained the weight loss goal of 7% (*n* = 32). To evaluate potential predictors of response in the outcome variables during weight loss, multiple linear regression was used to evaluate the independent factors associated with change with both pulse wave velocity and AIX75, aortic blood pressure and brachial blood pressure due to the intervention.

A portion of the full study sample (*N* = 16) was randomized into the PA-REC (*n* = 8) and the WM-REC groups (*n* = 8) during the weight maintenance phase (see consort diagram Figure 1). The loss of participants was primarily due to the coronavirus pandemic (*n* = 12). Participants disenrolled for safety purposes and one participant was early-termed (~4 weeks early), and therefore did not have appropriate exercise data for the present analysis. A Student's *t*-test was used to evaluate differences between the PA-REC (*n* = 8) and WM-REC (*n* = 8) groups at baseline and the beginning of the weight maintenance phase. An analysis of covariance was used to evaluate the difference in the change in outcome measures from the end of the weight loss phase (week 10) to follow-up (week 28), with adjustments for the variable baseline value at week 10 value and change in the outcome measure during the weight loss phase. Pearson correlations were used to evaluate relationships between the change in vascular stiffness and other variables. Stepwise linear regression was used to evaluate for independent predictors of arterial stiffness, aortic blood pressure, and brachial blood pressure measures. An alpha level of *p* < .05 was used as the criteria for significance testing for all statistical tests.

## 3 ∣ RESULTS

Baseline participant characteristics are displayed in Table 1. The sample (*N* = 35) had a mean (SD) age of 47.5 (40.4) years, a mean BMI of 34.1 (3.4) kg/m^2^, and a mean cfPWV of 7.8 (1.5) m/s. Baseline cfPWV was associated with age (*r* = 0.60, *p* < .001), brachial systolic blood pressure (*r* = 0.50, *p* = .002), brachial resting diastolic pressure (*r* = 0.49, *p* = .003), aortic systolic blood pressure (*r* = 0.51, *p* = .002), and aortic diastolic blood pressure (*r* = 0.50, *p* = .002), but not weight, body composition, or other cardiometabolic risk factors (*p* > .05). AIX75 was associated with absolute fitness (*r* = −0.35, *p* = .039), relative fitness (*r* = −0.37, *p* = .027), resting heart rate (*r* = 0.37, *p* = .028), and absolute fat mass (*r* = 0.39, *p* = .020).

### 3.1 ∣ Weight loss phase

The percent weight loss following the intervention was 9.4% with 91.4% of participants meeting the goal of 7% weight loss. In terms of clinically meaningful thresholds for weight loss, 2.9% of the sample achieved less than 5% weight loss, 65.7% between 5%–10%, and 31.4% of the sample achieved 10% or greater weight loss. The attendance rate for behavioral classes was 84.9% and the mean adherence to the aerobic exercise training was 94.0%. Further details about the fidelity of the exercise training intervention are shown in (see Tables S1-S4). The changes in weight, body composition and cardiometabolic outcome measures are shown in Table 2. We observed significant improvements in weight, waist circumference, body fat percentage, relative fitness, estimated METs, blood lipids (LDL, triglycerides, total cholesterol), glucose, insulin, and HOMA-IR levels (all *p*s <.001), but not absolute fitness (*p* = .369). We also observed a small reduction in HDL cholesterol after the intervention (*p* = .033).

**FIGURE 2:**
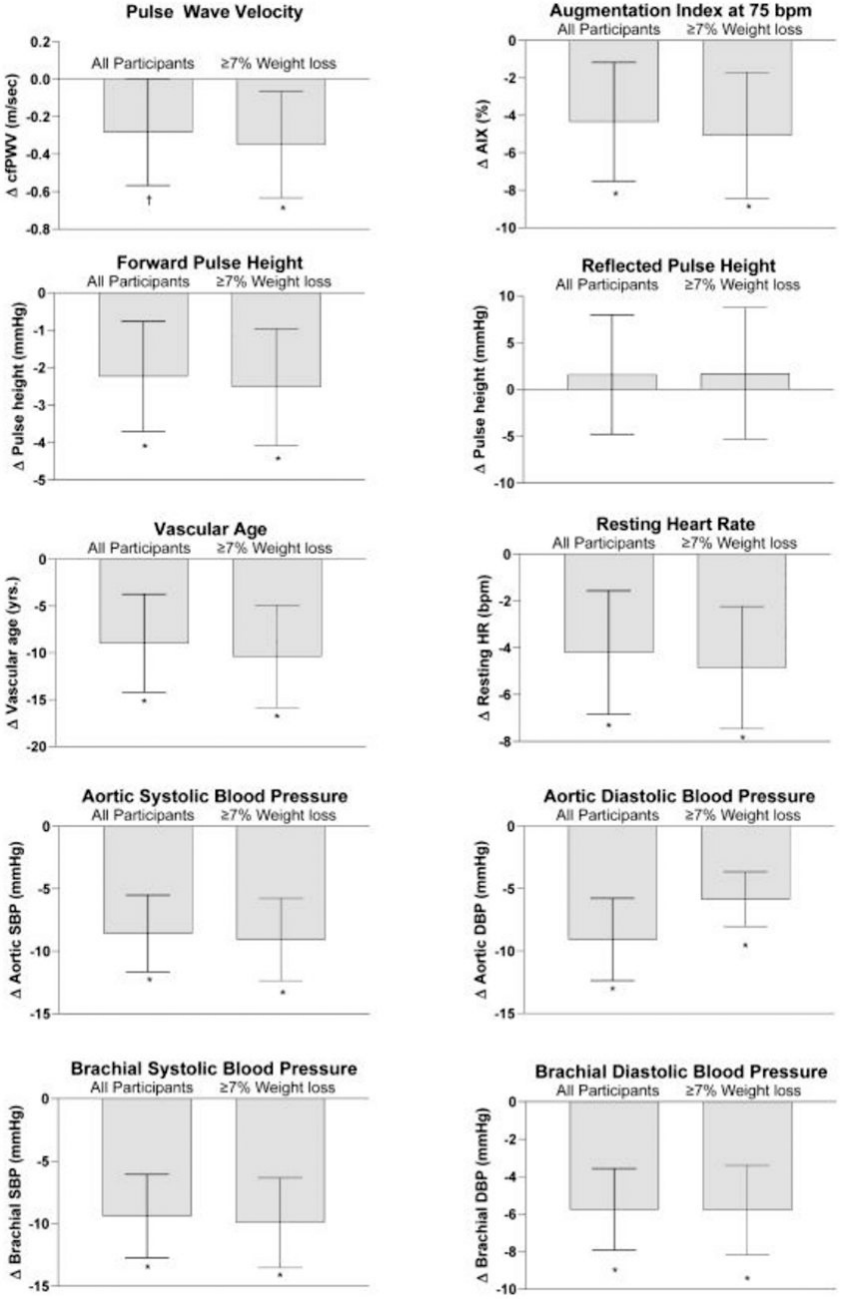
The change in arterial stiffness, blood pressure, and vascular factors during the weight loss phase.

The changes in vascular variables during the weight loss phase are shown in Figure 2. Briefly, we observed a significant improvement in AIX75 forward pulse height, resting brachial blood pressure, aortic blood pressure, and vascular age (all *p*s <.05). The reduction in pulse wave velocity approached significance in the entire study sample (*p* = .051). When the sample was restricted to participants who achieved clincially significant weight loss of ≥7% (*n* = 32), there was a significant reduction in pulse wave velocity (*p* = .018). There were no significant changes in the reflected pulse height or reflection magnitude in participants who made the weight loss goal or the entire study sample during the weight loss phase (*p* > .05).

Linear regression models were used to evaluate the independent predictors associated with the change in arterial stiffness and other related variables (Tables S1-S4). Change in pulse wave velocity was inversely associated with baseline pulse wave velocity (*r*^2^ = 0.43, *p* < .001), but positively associated with average exercise time (*r*^2^ = 0.14, *p* = .004). Change in AIX75 was negatively associated with baseline AIX75 (*r*^2^ = 0.16, *p* = .024), but positively associated with a change in weight (*r*^2^ = 0.16, *p* = .05). Change in aortic systolic blood pressure was negatively associated with baseline aortic systolic blood pressure (*r*^2^ = 0.35, *p* = .001). Change in aortic diastolic blood pressure was negatively associated with baseline aortic diastolic blood pressure (*r*^2^ = 0.31, *p* < .001) and exercise adherence during the weight loss phase (*r*^2^ = 0.09, *p* = .050).

### 3.2 ∣ Weight maintenance phase

The characteristics of the sample of participants who completed the maintenance phase (*n* = 16) are shown in Tables S1-S4. This sample had a mean (SD) age of 52.1 (8.2) years, a mean BMI of 30.4 (3.2) kg/m^2^, was 88.2% female and 25.0% African American. At the beginning of the weight maintenance phase, participants in the WM-REC group had higher pulse wave velocity, brachial diastolic blood pressure, aortic blood pressure, and total cholesterol levels (*p* < .05), but not AIX75 (*p* = .282). However, no significant differences were observed for the change in outcome variables during the weight loss phase between participants in the PA-REC and the WM-REC groups in any of the outcome variables (all *p*s >.05).

The exercise training characteristics for the weight maintenance phase are shown in Table 3A. There were no significant differences between groups in exercise adherence, compliance, mean training heart rate, and mean training percent peak VO_2_ (all *p*s >.05). By design, the number of exercise sessions and total minutes of aerobic exercise per week were greater in the WM-REC group compared to the PA-REC group (all *p*s <.05).

**FIGURE 3:**
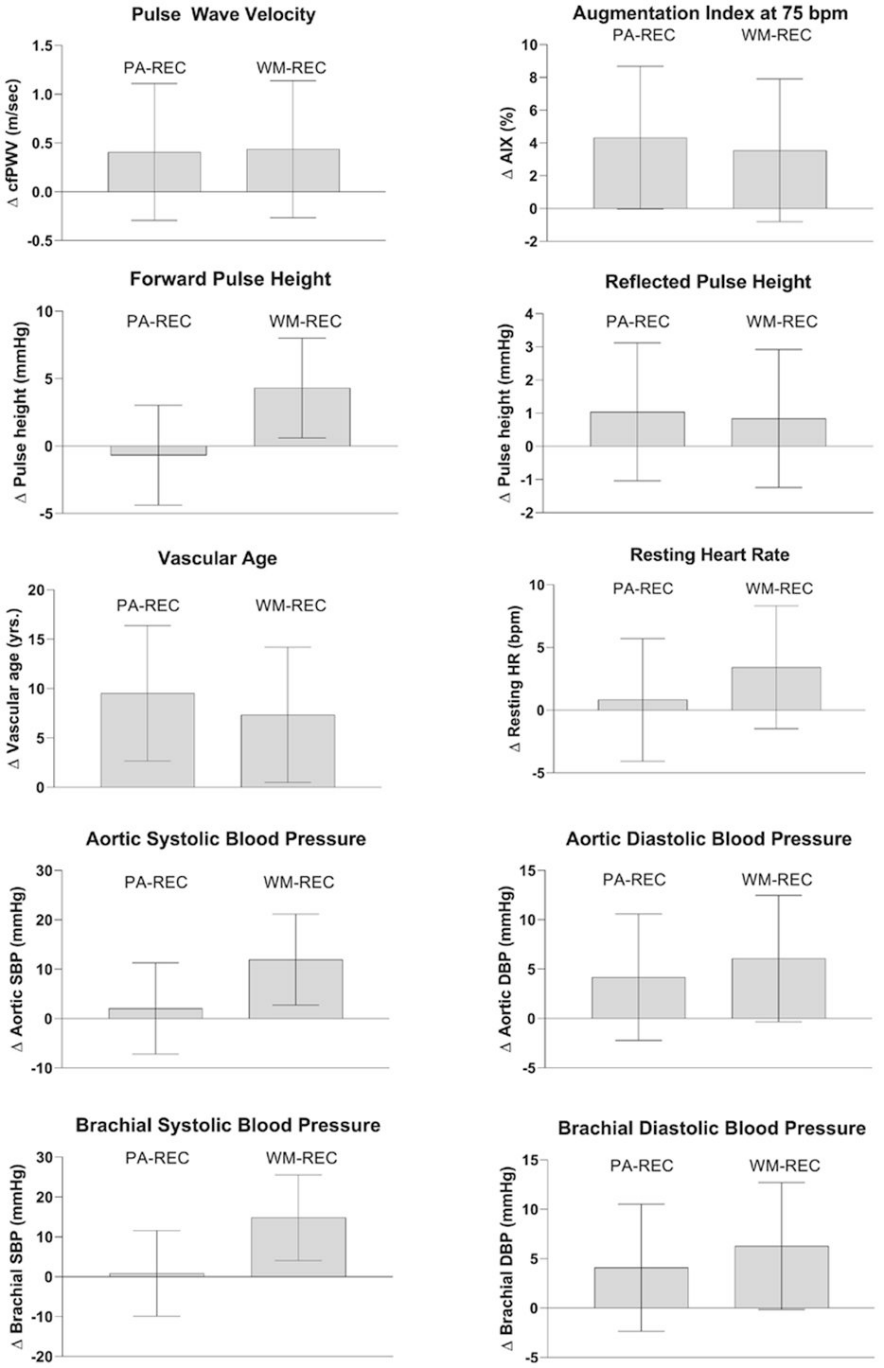
The change in arterial stiffness, blood pressure, and vascular factors during the weight maintenance phase.

The changes in outcome measures during the weight maintenance phase are shown in Figure 3 and Table 3B. For arterial stiffness measures, there were no group differences in the change in pulse wave velocity, AIX75, brachial blood pressure, aortic blood pressure or vascular age between groups (all *p*s >.05). In addition, no group differences were observed for changes in weight, body composition, and glucose metabolism variables (all *p*s >.05). However, there was a larger increase in estimated METs in the WM-REC (1.6 METs) compared to the PA-REC group (0.10 METs) (*p* = .03). Similarly, the change in absolute fitness between the PA-REC (0.06 L/min) and the WM-REC group (0.15 L/min) approached significance (*p* = .10). In addition, there was a larger increase in total cholesterol in the PA-REC group compared to the WM-REC group (*p* = .04). There were within-group increases in vascular age (*p* = .01) and HDL cholesterol (*p* = .001) in the PA-REC group. Similarly, there were within-group increases in vascular age (*p* = .03), aortic systolic blood pressure (*p* = .01), brachial blood pressure (*p* = .01), and HDL cholesterol (*p* = .04) in the WM-REC group.

Linear regression models evaluating independent factors associated with changes in arterial stiffness variables during weight maintenance are shown in Tables S1-S4. The change in pulse wave velocity was associated with a change in LDL cholesterol (*r*^2^ = 0.45, *p* = .004) and mean aerobic training VO_2_ (*r*^2^ = 0.17, *p* = .033). Change in AIX75 was associated with the change in AIX75 during the weight loss phase (*r*^2^ = 0.29, *p* = .03) and change in LDL (*r*^2^ = 0.29, *p* = .01). Change in aortic systolic blood pressure was associated only with mean aerobic training VO_2_ (*r*^2^: 0.37 *p* = .013). Change in aortic diastolic blood pressure was associated with mean aerobic training VO_2_ (*r*^2^ = 0.53, *p* < .001) and change in triglycerides (*r*^2^ = 0.13, *p* = .04). Change in brachial systolic blood pressure was associated with aerobic training VO_2_ (*r*^2^ = 0.30, *p* = .03), weight change during the weight maintenance phase (*r*^2^ = 0.20, *p* = .034), and change in triglycerides (*r*^2^ = 0.16, *p* = .04).

## 4 ∣ DISCUSSION

The primary findings of the present study are that clinically significant weight loss (≥7%) and aerobic exercise training resulted in improvement in measures of arterial stiffness, wave reflection and blood pressure. In addition, during the weight maintenance phase, the major factors that were associated with changes in both arterial stiffness and blood pressure parameters were increases in lipids (LDL and triglycerides) and aerobic exercise training at a higher intensity. Our findings are novel as this is the first study to our knowledge that has evaluated the impact of aerobic exercise training during weight loss and weight maintenance on arterial stiffness, which is an independent risk factor for cardiovascular mortality.^39^

The weight loss phase, composed of an OPTIFAST diet and aerobic exercise training, showed improvements in pulse wave velocity of 0.34 (m/s) in participants that achieved the weight loss goal of at least 7%. Based on a recent meta-analysis, this change corresponds with a 4.1% improvement in cardiovascular disease risk [56]. The magnitude of change in pulse wave velocity in the present study agrees with other published lifestyle interventions. For example, Nordstrand et al.^40^ observed that a 7-week intensive nutritional weight loss program combined with two 90-min exercise sessions per week improved cfPWV by −0.4 m/s in Norwegian adults with class II obesity (BMI between 35–40 kg/m^2^). Similarly, Blumenthal et al.^41^ observed that a 4-month program of the DASH diet with aerobic training (three sessions, 90 min total per week) resulted in a reduction of −0.65 m/s in cfPWV. The present study expands these findings to the OPTIFAST diet and supports its efficacy to improve arterial stiffness and blood pressure in adults who are overweight and with obesity.

During the weight loss phase, baseline cfPWV was the strongest predictor for the change in cfPWV (approximately 43% of the variation). While an important limitation of this analysis is that it may be vulnerable to regression to the mean effects,^42^ our results suggest that the weight loss phase improved pulse wave velocity in participants with the highest risk of CVD at baseline based on arterial stiffness values. Comparably, Barinas-Mitchell^22^ observed that baseline value was the strongest predictor of change in pulse wave velocity (*r*^2^ = 0.77) in adults with obesity and type 2 diabetes. In the present study, we observed that neither weight loss nor fat loss were independent predictors for the change in arterial stiffness, which may be due to the limited range of weight loss across participants (most participants lost around 5%–12% weight loss). The mean percent weight loss was 9.4% in PREVAIL-P, so it is unlikely that weight loss did not play some role in the improvements in vascular adaptations, as weight loss improves many factors associated with elevated pulse wave velocity (e.g., dyslipidemia, high inflammation, hyperglycemia, hypertension).^43-45^ It is plausible, therefore, that the relationship between weight loss and cfPWV is more apparent across a wider distribution of weight loss.^22^ The lack of a linear relationship between weight loss and change in cfPWV in PREVAIL-P is in agreement with published studies^22,46^ and the results of a meta-analysis of weight loss interventions.^21^ Another vascular factor that improved during the weight loss phase was augmentation index, which is an independent risk factor for cardiovascular mortality. The reduction in augmentation index observed in our sample (~5%) is associated with a 19% reduction in cardiovascular events.^47^

During the weight maintenance phase, we observed no significant differences in the change in cfPWV between the level of exercise at the minimum physical activity guidelines and the weight maintenance guidelines following clinically significant weight loss. Based on linear regression models, increases in cfPWV during the maintenance period were associated with increases in LDL and lower aerobic training intensities (both groups combined). These findings are supported by published data suggesting that higher levels of dyslipidemia are associated with elevated pulse wave velocity.^16,48^ LDL levels, specifically, have been independently associated with worsened pulse wave velocity in some studies.^16,48^ In addition, high-intensity aerobic exercise training has been shown to improve arterial stiffness and other factors that affect stiffness (e.g., endothelial function,^49^ increases in cardiorespiratory fitness,^28^ wall-remodelling via increases in metalloproteinases,^50^ etc.) to a greater extent than moderate intensity training. Similarly, in this study, AIX75 was also associated with changes in LDL and changes in weight. This suggests that maintaining optimal lipid levels after weight loss may be especially important in maintaining improvements in arterial stiffness during weight maintenance. Future studies should evaluate the impact of high-intensity aerobic exercise training combined with a lipid-focused dietary program during the weight maintenance period on arterial stiffness and blood pressure parameters.

Strengths of the present investigation include that the PREVAIL-P study had supervised aerobic training sessions during the entire study (weight loss and weight maintenance phases), pulse wave velocity was captured using cfPWV (the gold standard method),^13^ and the aerobic exercise levels selected during the weight maintenance period were associated with established guidelines for physical activity and weight maintenance. Limitations of the present study are that we included overweight to class II obesity, therefore our results do not extend to individuals in the class III obesity range, who likely have worsened arterial stiffness compared to the present sample. In addition, our sample was composed of mostly females, so our results may have limited generalizability to males. In addition, augmentation index and central blood pressure measures were performed in the seated position as opposed to the supine position. However, there are also published data evaluating central blood pressure and PWA with CVD risk,^51-54^ cross-sectional studies with CVD risk factors,^55-57^ and an exercise intervention^58^ when taken in the seated position. Lastly, a portion of the sample was lost due to the coronavirus pandemic during the weight maintenance phase. Therefore, our results should be interpreted with some caution and replication in future studies may be warranted.

In conclusion, the results of the present study suggest that clinically significant weight loss, along with aerobic exercise training, improved arterial stiffness, blood pressure, and a variety of other cardiometabolic factors. However, during the weight maintenance period, we did not observe significant changes in cfPWV at aerobic exercise levels consistent with the physical activity guidelines or weight maintenance guidelines. However, exercise intensity and changes in LDL cholesterol during the maintenance period were independent predictors of response. Future studies should investigate if aerobic exercise at higher intensity, while limiting increases in lipids, is a potential intervention strategy to maintain improvements in arterial stiffness after clinically significant weight loss.

## Supplementary Material

Supplementary Material

## Figures and Tables

**TABLE 1:** Baseline participant characteristics. Continuous baseline data is presented in means (SD) and categorical data is presented in % (*n*).

Variable	(*N* = 35)
Age (years)	47.5 (10.4)
Female % (*n*)	80.0 (28)
African American % (*n*)	35.3 (12)
Weight (kg)	95.5 (12.4)
Body mass index (kg/m^2^)	34.1 (3.4)
Body fat (%)	41.7 (5.7)
Waist circumference (cm)	98.0 (9.6)
Peak VO_2_ (L/min)	21.3 (4.2)
Peak VO_2_ (ml/kg/min)	2.0 (0.5)
Estimated METs	8.3 (1.2)
Resting heart rate (bpm)	67.7 (8.6)
Brachial systolic blood pressure (mmHg)	122.8 (13.6)
Brachial diastolic blood pressure (mmHg)	77.3 (9.0)
Aortic systolic blood pressure (mmHg)	113.7 (12.1)
Aortic diastolic blood pressure (mmHg)	78.0 (9.1)
Glucose (mg/dl)	96.4 (9.8)
Insulin (ulU/ml)	18.0 (10.8)
HOMA-IR	4.4 (2.9)
LDL cholesterol (mg/dl)	113.4 (27.7)
HDL cholesterol (mg/dl)	52.7 (12.9)
Total cholesterol (mg/dl)	187.6 (30.4)
Triglycerides (mg/dl)	107.1 (55.7)
Forward pulse height (mmHg)	25.3 (4.1)
Backward pulse height (mmHg)	16.1 (2.8)
Reflection magnitude (%)	63.8 (10.6)
Vascular age (years)	52.5 (14.7)
Pulse wave velocity (m/s)	7.8 (1.5)
Augmentation index at 75 bpm (%)	27.4 (8.9)

**TABLE 2:** Changes in cardiometabolic risk factors following the weight loss intervention. Results are displayed in mean change with 95% confidence intervals.

	Entire sample (*N* = 35)		Participants that achieved the weight loss goal (*N* = 32)	
Variable (*N* = 35)	Δ (95% CI)	*p*-value	Δ (95% CI)	*p*-value
Δ Weight (kg)	−8.9 (−10.1 to −7.8)	<.001	−9.2 (−10.4 to −8.1)	<.001
Δ Waist circumference (cm)	−8.5 (−10.3 to −6.8)	<.001	−8.5 (−10. to −1.5)	<.001
Δ Body fat (%)	−2.0 (−2.6 to −1.4)	<.001	−2.1 (−2.7 to −1.5)	<.001
Δ Peak VO_2_ (l/min)	0.02 (−0.02 to 0.075)	.369	0.020 (−0.04 to 0.08)	.466
Δ Peak VO_2_ (ml/kg/min)	2.5 (1.9 to 3.0)	<.001	2.6 (1.3 to 2.1)	<.001
Δ Estimated METs	0.8 (0.5 to 1.2)	<.001	0.7 (0.7 to 1.2)	<.001
Δ Glucose (mg/dl)	−11.1 (−13.9 to −8.4)	<.001	−11.2 (−14.1 to −8.2)	<.001
Δ Insulin (ulU/ml)	−10.7 (−13.3 to −8.3)	<.001	−10.5 (−13.2 to −7.9)	<.001
Δ HOMA-IR	−2.8 (−3.5 to −2.1)	<.001	−2.7 (−3.4 to −2.0)	<.001
Δ LDL cholesterol (mg/dl)	−7.5 (13.0 to −1.9)	.001	−8.3 (−14.3 to −2.4)	.008
Δ HDL cholesterol (mg/dl)	−2.5 (−4.9 to −0.21)	.033	−2.6 (−5.1 to −0.0)	.050
Δ Total cholesterol (mg/dl)	−14.9 (−12.5 to −8.4)	<.001	−16.0 (15.9 to 26.3)	<.001
Δ Triglycerides (mg/dl)	−23.8 (−37.5 to −10.1)	.001	−25.1 (−40.0 to −10.2)	.002

**TABLE 3:** Change in outcome variables during the weight maintenance phase. (A) Intervention fidelity measures in the PA-REC and the WM-REC groups. Variables are expressed in mean (SD). (B) Changes in cardiometabolic body composition, fitness and lipid variables during the weight maintenance phase in mean (SD).

(A) *Exercise training variables*	PA-REC (*N* = 8)	WM-REC (*N* = 8)	*p*-value
Exercise sessions (per week)	2.6 (0.4)	3.5 (0.6)	.004
Exercise adherence (%)	95.3 (11.5)	88.1 (11.5)	.19
Exercise compliance (%)	91.7 (11.5)	86.4 (12.9)	.40
Exercise time per week (min)	105.2 (17.2)	160.1 (18.2)	<.001
Total exercise time (min)	1904.7 (301.6)	2901.3 (329.9)	<.001
Mean training HR (bpm)	116.4 (11.5)	116.7 (10.8)	.88
Percent VO_2_ (%)	61.8 (5.5)	61.0 (5.8)	.74
(B) *Cardiometabolic variables*			
Δ Weight (kg)	−0.1	−1.7	.49
Δ Weight loss (%)	−0.1	−1.5	.56
Δ Body mass index (kg/m^2^)	−0.2	−0.5	.67
Δ Body Fat (%)	−1.3	−1.3	.97
Δ Visceral fat (g)	−18.0	−32.8	.67
Δ Waist circumference (cm)	−0.8	−2.3	.40
Δ Peak VO_2_ (l/min)	0.06	0.15	.11
Δ Peak VO_2_ (ml/kg/min)	0.78	2.2	.14
Δ Estimated METs	0.10	1.6	.03
Δ Brachial systolic blood pressure (mmHg)	0.81	14.8	.13
Δ Brachial diastolic blood pressure (mmHg)	4.1	6.3	.64
Δ Glucose (mg/dl)	2.7	2.2	.87
Δ Insulin (ulU/ml)	0.6	3.4	.37
Δ HOMA-IR	0.1	0.7	.34
Δ LDL cholesterol (mg/dl)	9.3	3.8	.69
Δ VLDL cholesterol (mg/dl)	0.7	2.9	.49
Δ HDL cholesterol (mg/dl)	12.0	6.8	.22
Δ Total cholesterol (mg/dl)	38.0	3.1	.04
Δ Triglycerides (mg/dl)	3.4	14.4	.49

## What is already known about this subject

- Arterial stiffness is an independent risk factor for cardiovascular mortality.
- Obesity is associated with an increased risk of arterial stiffness.
- Weight loss and exercise are known to improve arterial stiffness.

## What this study adds

- While weight loss is known to improve arterial stiffness, few studies have evaluated the impact of exercise level on weight maintenance.
- Factors that predict changes in arterial stiffness have not previously been evaluated during weight maintenance.
