# Supplementary material for: The effect of exercise training level on arterial stiffness after clinically significant weight loss
Source: Clin Obes. Author manuscript; Available in PMC 2024 Apr 23. (PMC11036368; doi:10.1111/cob.12584)
Supplement: Supplementary Material [file NIHMS1985555-supplement-Supplementary_Material.pdf]

Supplementary Information:

The Effect of Exercise Training Level on Arterial Stiffness after Clinically Significant Weight Loss

Damon L. Swift [1,2,†], Joshua E. McGee [1,2], Emily E. Grammer [1,2,†], Anna C. Huff [1,2], Marie C. Clunan [1,2], Nicole Gniewek [1,2], Taylor T. Brown [1,2], Briceida G. Osbourne [7], Joseph A. Houmard [1,2], Robert A. Carels [3], Walter J. Pories [5], Laura E. Matarese [4]

Department of Kinesiology [1], Human Performance Laboratory [2], Department of Psychology [3], Department of Internal Medicine [4], Department of Surgery [5], East Carolina Diabetes and Obesity Institute [6], East Carolina University Greenville, NC, 27858; Vidant Wellness Center [7], Greenville NC, 27858. [8]. † Current address: Department of Kinesiology, University of Virginia, Charlottesville, VA 22904

Supplemental Table 1. Intervention statistics. Exercise intervention fidelity data from the exercise training intervention. Data are **presented in mean (SD).**

	**Week 1**	**Week 2**	**Week 3**	**Week 4**	**Week 5**	**Week 6**	**Week 7**	**Week 8**	**Week 9**	**Week 10**
Mean frequency (times per week)	2.0 (0.4)	2.4 (0.5)	2.4 (0.5)	2.6 (0.6)	2.7 (0.5)	2.6 (0.7)	2.6 (0.5)	2.7 (0.5)	2.6 (0.6)	3.0 (1.0)
Mean speed (mph)	2.8 (0.3)	2.9 (0.3)	3.0 (0.3)	3.0 (0.3)	3.1 (0.3)	3.2 (0.5)	3.1 (0.3)	3.1 (0.3)	3.2 (0.3)	3.2 (0.3)
Mean grade (%)	1.5 (0.8)	1.7 (0.8)	1.9 (0.8)	2.1 (0.8)	2.2 (0.8)	2.5 (0.7)	2.6 (0.7)	3.0 (0.6)	3.1 (0.5)	3.2 (0.5)
Mean exercise volume (MET minutes)	199.0 (36.3)	334.5 (55.5)	372.3 (62.4)	441.9 (57.7)	480.2 (67.8)	495.2 (129.6)	533.1 (113.6)	602.1 (112.2)	621.9 (148)	712.2 (242.3)
Mean exercise time (min)	53.0 (12.2)	86.6 (16.2)	92.6 (15.6)	105.5 (20.2)	113.0 (24.2)	111.2 (34.2)	117.2 (30.4)	127.0 (29.6)	126.1 (33.0)	145.1 (55.5)
Mean energy expenditure (kcals)	314.4 (78.0)	538.9 (115.0)	576.1 (158.1)	663.3 (157.9)	746.3 (166.1)	759.5 (202.7)	821.6 (198.8)	918.6 (215.5)	934.0 (246.6)	1055.1 (372.0)
Mean estimated intensity (%VO_2)	62.1 (9.2)	63.9 (8.7)	62.6 (7.0)	63.5 (6.1)	63.1 (5.7)	62.4 (4.9)	62.6 (5.2)	62.9 (6.7)	63.2 (5.6)	62.6 (7.6)
Mean heart rate (bpm)	118.6 (17.1)	120.7 (17.1)	119.8 (12.1)	120.1 (12.8)	119.5 (11.9)	119.6 (13.9)	118.9 (12.0)	119.2 (12.3)	119.4 (13.2)	118.6 (12.8)
Mean exercise adherence (%)	99.5 (18.1)	95.6 (15.9)	93.1 (15.6)	98.2 (12.8)	96.0 (13.6)	90.0 (23.6)	88.8 (18.9)	92.6 (17.3)	88.8 (21.3)	101.7 (34.6)
Mean exercise compliance (%)	97.1 (16.9)	96.2 (10.8)	93.8 (14.1)	96.7 (11.3)	94.3 (12.7)	90.2 (22.5)	87.9 (18.0)	91.4 (16.8)	87.3 (20.1)	101.4 (30.9)
Mean exercise dose (KKW)	3.3 (0.7)	5.9 (1.0)	6.3 (1.6)	7.4 (1.6)	8.3 (1.3)	8.7 (2.3)	9.4 (1.9)	10.6 (1.9)	10.9 (2.6)	12.4 (4.2)

Supplemental Table 2. Demographic characteristics for participants at the beginning of the weight maintenance phase. Continuous variables are described in mean (SD) and categorical variables are described using % (n). * Denotes significant difference (p<0.05)

**Variable**	**PA-REC (N=8)**	**WM-REC (N=8)**
Age (yrs.)	50.8 (10.0)	52.6 (6.6)
Female % (n)	87.5 (7)	87.5 (7)
African American % (n)	25.0 (2)	25.0 (2)
Weight (kg)	81.6 (13.3)	84.1 (14.4)
Body mass index (kg/m^2)	29.9 (3.7)	30.2 (2.8)
Visceral fat (g)	540.0 (198.2)	624.3 (131.0)
Body fat (%)	40.9 (5.8)	41.2 (3.8)
Waist circumference (cm)	86.2 (6.6)	91.3 (7.6)
VO_2 max (L/min)	1.9 (0.4)	1.9 (0.4)
VO_2 max (ml/kg/min)	23.0 (4.2)	22.9 (1.7)
Estimated METs	9.2 (1.3)	8.9 (0.9)
Resting heart rate (bpm)	67.0 (9.0)	68.5 (6.8)
Brachial systolic blood pressure (mmHg)	104.1 (8.2)	120.1 (10.9) *
Brachial diastolic blood pressure (mmHg)	66.9 (4.8)	72.6 (6.4)
Aortic systolic blood pressure (mmHg)	97.5 (7.6)	110.5 (9.1) *
Aortic diastolic blood pressure (mmHg)	67.3 (4.8)	73.3 (6.1) *
Glucose (mg/dL)	83.6 (5.1)	83.8 (7.8)
Insulin (uIU/mL)	4.2 (3.3)	5.6 (2.6)
HOMA-IR	0.9 (0.8)	1.2 (0.6)
LDL cholesterol (mg/dL)	97.0 (18.8)	121.8 (20.3) *
VLDL cholesterol (mg/dL)	16.7 (9.7)	15.6 (2.5)
HDL cholesterol (mg/dL)	52.1 (18.7)	54.4 (8.9)
Total cholesterol (mg/dL)	165.8 (17.3)	191.8 (16.3) *
Triglycerides (mg/dL)	83.6 (48.2)	78.0 (12.4)
Forward pulse height (mmHg)	21.4 (4.6)	25.1 (4.4)
Backward pulse height (mmHg)	13.3 (2.6)	15.9 (3.7)
Reflection magnitude (%)	63.4 (13.4)	62.9 (7.4)
Vascular age (yrs.)	39.6 (13.8)	41.1 (14.2)
Pulse wave velocity (m/sec)	7.0 (0.7)	8.2 (0.9) *
Augmentation index at 75 bpm (%)	23.6 (5.9)	19.6 (7.9)

Supplemental Table 3. Linear regression models for the change in arterial stiffness and related vascular parameters during the weight loss phase. cfPWV: cartoid-to-femoral pulse wave velocity, SBP: systolic blood pressure, DBP: diastolic blood pressure, WL: weight loss.

Variable	β	Variables	Partial r^2	p-value
**Δ Pulse wave velocity (m/sec)**<br>(model r^2: 0.57)	-0.40<br>0.02	Baseline cfPWV<br>Mean exercise time	0.43<br>0.14	<0.001<br>0.044
**Δ Augmentation index at 75 bpm (%)**<br>(model r^2: 0.28)	-0.52<br>1.00	Baseline AIX75<br>Δ Weight	0.16<br>0.27	0.024<br>0.046
**Δ Aortic systolic blood pressure (mmHg)**<br>(model r^2: 0.35)	-0.45	Baseline aortic DBP	0.35	<0.001
**Δ Aortic diastolic blood pressure (mmHg)**<br>(model r^2: 0.40)	-0.45<br>-0.24	Baseline aortic DBP<br>Exercise adherence WL	0.32<br>0.09	<0.001<br>0.050
**Δ Brachial systolic blood pressure (mmHg)**<br>(model: r^2: 0.43)	-0.43<br>0.41	Baseline systolic BP<br>Δ HDL	0.35<br>0.09	<0.001<br>0.045
**Δ Brachial diastolic blood pressure (mmHg)**<br>(model r^2: 0.31)	-0.42	Baseline diastolic BP	0.31	<0.001

Supplemental Table 4. Stepwise linear regression models for the change in arterial stiffness and blood pressure variables during weight maintenance. Variables entered into the model: Mean exercise times, amount of exercise sessions per week, exercise adherence, mean training VO_2, baseline level, change in variable during the weight loss phase, change in estimated METs, change in absolute VO_2, change in relative VO_2, change in visceral fat, change in LDL, change in HDL, change in non-HDL, change in triglycerides, change in weight, and change in body fat.

Variable	β	Variables	Partial r^2	p-value
**Δ Pulse wave velocity (m/sec)**	0.03	Δ LDL	0.45	0.004
(model r^2: 0.61)	-0.05	Mean training %VO_2	0.17	0.033
**Δ Augmentation index at 75 (%)**	-0.44	Δ AIX in weight loss	0.29	0.031
(model r^2: 0.58)	0.17	Δ LDL	0.29	0.010
**Δ Aortic systolic blood pressure (mmHg)**	-1.30	Mean training %VO_2	0.36	0.013
(model r^2: 0.36)				
**Δ Aortic diastolic blood pressure (mmHg)**	-1.11	Mean training %VO_2	0.53	0.002
(model r^2: 0.66)	-0.10	Δ Triglycerides	0.13	0.044
**Δ Brachial systolic blood pressure (mmHg)**	-1.90	Mean training %VO_2	0.30	0.029
(model: r^2: 0.66)	-0.20	Δ Triglycerides	0.21	0.035
	-1.72	Δ Weight	0.16	0.035
**Δ Brachial diastolic blood pressure (mmHg)**	-1.13	Mean training %VO_2	0.59	0.001
(model r^2: 0.68)	-0.10	Δ Triglycerides	0.12	0.047
